# How the Problem Of the Blind Can Be Solved

**Published:** 1917-09-22

**Authors:** 


### HOW THE PROBLEM OF THE BLIND CAN BE SOLVED.

### The Report of the Departmental Committee.

According to the Census returns of 1911 there were 33,965 blind in the United Kingdom. These persons were almost equally divided between the two sexes, more than half being over fifty-five years of age. Comparing the figures for 1911 with those for 1901, it is discovered that while the total number of blind has increased, the proportion of blind to population has diminished, except in Ireland; the present proportion in the United Kingdom is 1 in 1,274. The Census returns, however, cannot be considered as entirely reliable, as there is no generally accepted definition of blindness; and it is probable that there must be an additionally large number of persons who, not being totally blind, are sufficiently incapacitated for work that can be carried out by a person with ordinary sight.

### Past Neglect.

The Departmental Committee for the Welfare of the Blind, appointed in 1914 by the President of the Local Government Board, has just issued its Report. The Committee was under the chairmanship of the Right Hon. W. Hayes Fisher, M.P., and included several well-known people either blind themselves or who have done work specially in organisations for the welfare of the blind. One of the members, Sir Arthur Pearson, Bart., is the President of the National Institute for the Blind and Chairman of the Blinded Soldiers and Sailors Care Committee, who are responsible for the well-known and successful work at St. Dunstan's Hostel. In an interview given to a newspaper representative Sir Arthur expressed the opinion that practically nothing has been done for the blind by the Government in the past. In the future Sir Arthur hoped that this regrettable condition of affairs will be replaced by one which will show that the State is prepared to undertake the responsibility of assisting those of its citizens whom in most cases the handicap of blindness deprives of the power of helping themselves to the fullness of their capacity. He pointed out that blind people do not as a rule wish to eat the bread of charity, and desire to be assisted to overcome a handicap which has been laid upon them, and to do their share of the work of the world. The example of St. Dunstan's was put forward to illustrate what can be done in the way of equipping blind people to become useful citizens, and to afford them the gratification of feeling that they are usefully occupied, that they are not blind in the ordinary acceptation of the term, but just normal people who cannot see and who have overcome that deficiency sufficiently to take their places in a busy world.

The Report of the Departmental Committee is exhaustive in character and contains much useful and interesting information, particularly in respect of the occupations chiefly followed by the blind, and of the incidence and causes of blindness. One of these causes, as is well known, is ophthalmia neonatorum, a form of inflammation affecting the conjunctiva in the newly born, occurring as a rule during the first days of life. It is nearly always due to infection of the eyes during birth, and if not treated promptly there is often grave danger of total blindness following. As might be expected, the Report emphasises the importance of the part played by the midwife in these cases, and a specialist who gave evidence before the Committee expressed a decided opinion that the present instructions to midwives are inadequate. It will be remembered that the Report of the Royal Commission on Venereal Diseases stated that as high a percentage as 25 per cent. of all cases of blindness has been attributed to gonorrhœal ophthalmia, and this opinion has been borne out by the inquiries of the Committee on the Welfare of the Blind. Naturally, the improvement in the status of the midwife must have a rapid and direct bearing on the statistics of blindness in the newly born.

Another form of blindness which is also largely preventable is that caused by mechanical injuries to persons following certain occupations such as bottling of aerated waters, several forms of granite working, etc.; a large number of these industrial accidents might be prevented by the wearing of suitable goggles.

### Hope for the Future.

On the whole, having regard to the steady increase in medical and surgical knowledge and the constant desire of Parliament to improve industrial conditions, it may with a certain amount of confidence be predicted that the number of blind will continue to decrease proportionately in relation to the total population. In any case the total number is relatively not a large one, and, regarded from a financial point of view, if the State were entirely to take over their care, the cost, after our education of the last few years in national expenditure, would not seem alarming. At present the total expenditure of the charities in the British Isles devoted to the welfare of the blind is about £250,000 annually. The Departmental Committee considers that by an additional contribution of the Government of about a quarter of a million, and a capital expenditure of £500,000, the whole problem would be solved.

### The Committee's Recommendations.

Taken in detail the recommendations of the Committee are as follows:—That a State Department, whose function shall be the general care and supervision of the blind, shall be set up in the Ministry of Health, whenever such a Ministry is created, and in the meantime it shall be set up in the Local Government Board. The central authority shall have at its disposal funds provided by the Exchequer in order to make grants, capital and annual, for the purposes indicated in the recommendations, and for such other purposes as may appear necessary in the course of its administration.

In respect of education the following recommendations are made:—

(a) The attention of elementary education authorities should be drawn to the imperative necessity of seeing that all possible steps are taken to discover the aptitudes of blind pupils.

(b) Residential institutions should be regarded as preferable to day centres for the majority of young children.

(c) The employment of blind teachers wherever practicable should be encouraged, and the salaries of blind teachers should be on an equality with those of sighted teachers.

(d) The Committee have no doubt that the Education Departments will give their closest attention to the need for Braille books, which the National Institute for the Blind is endeavouring to meet.

(e) The education authorities should take steps to increase the number of schools or classes for the separate treatment of myopic and partially sighted children.

(f) The provision of a system of public elementary education in Ireland should be established at the earliest opportunity.

(g) A uniform scheme of after-care should be initiated in the elementary educational system.

(h) We recommend for consideration whether the education of blind children in institutions in Scotland might possibly be facilitated if it were made a charge on the Central Education Fund.

Inadequate Workshop Accommodation.

The Committee expresses the opinion that the crux of the problem of the blind in the United Kingdom is the inadequacy of workshop accommodation. The following recommendations are amongst those submitted:—

(a) The existing accommodation should be at least doubled, and in the first instance this provision might be effected by the extension of existing workshops.

(b) The central authority should make arrangements with the spending departments of the Government to secure that in certain contracts blind institutions are given the preference.

(c) The central authority should take steps to eliminate the unnecessary competition between existing institutions by securing the amalgamation or affiliation of the small workshops to the larger institutions, special attention being given to London.

(d) The central authority should also have funds at its disposal to make grants for the assistance of the certified institutions and organisations; and in making such grants should take steps to establish schemes whereby a minimum wage would be secured.

(e) The care of persons working at home should receive the careful attention of the central authority.

(f) The central authority should direct particular attention to the employment of blind women.

Much public sympathy has been awakened by the lot of our sailors and soldiers who have lost their sight while fighting for the country, and this sympathy has naturally been extended to the blind generally, as is indicated by an increase in the public support of existing institutions. There never was a time when legislation framed to ameliorate the condition of the blind could be more popular, and it is to be hoped that the Report and recommendations of the Committee will commend themselves to our legislators, and cause them to lose no time in taking steps to make adequate provision for the care, treatment, and comfort of those who are so unfortunate as to lose one of the most precious of the senses.

